# Vascular Impulse Technology versus elevation in the treatment of posttraumatic swelling of extremity fractures: study protocol for a randomized controlled trial

**DOI:** 10.1186/s13063-017-1824-8

**Published:** 2017-02-16

**Authors:** Marc Schnetzke, Benedict Swartman, Isabel Bonnen, Holger Keil, Svenja Schüler, Paul A. Grützner, Jochen Franke

**Affiliations:** 1 Department for Trauma and Orthopaedic Surgery, BG Trauma Center Ludwigshafen at Heidelberg University Hospital, Ludwigshafen am Rhein, Germany; 2 Institute of Medical Biometry and Informatics, University of Heidelberg, Heidelberg, Germany

**Keywords:** Posttraumatic swelling, Elevation, Vascular impulse, Cryotherapy, Upper extremity, Lower extremity, Intermittent pneumatic compression

## Abstract

**Background:**

Fractures of the extremities are often complicated by a variable degree of swelling secondary to hemorrhage and soft tissue injury. Patients typically require up to 7 days of inpatient bed rest and elevation to reduce swelling to an acceptable level for operative treatment with internal fixation. Alternatively, an intermittent pneumatic compression device, such as the Vascular Impulse Technology (VIT) system, can be used at the injured extremity to reduce the posttraumatic swelling. The VIT system consists of a pneumatic compressor that intermittently rapidly inflates a bladder positioned under the arch of the hand or the foot, which results in compression of the venous hand or foot plexus. That intermittent compression induces an increased venous velocity and aims to reduce the soft tissue swelling of the affected extremity.

**Methods/design:**

The VIT study is a prospective, monocenter, randomized controlled trial to compare the VIT system with elevation in the treatment of posttraumatic swelling in the case of a fracture of the upper and lower extremity. This study will include 280 patients with fractures of the upper and the lower extremity with nine different injury types. For each of the nine injury types a separate randomization to the two intervention groups (VIT group or control group) will be performed. The primary outcome parameter is the time taken for the swelling to resolve sufficiently to permit surgery. A separate analysis for each of the nine injury types will be performed.

**Discussion:**

In the proposed study, the effectiveness of the VIT system in the treatment of posttraumatic swelling of upper and lower extremity fractures will be evaluated.

**Trial registration:**

German Clinical Trial Register, No. DRKS00010510. Registered on 17 July 2016.

**Electronic supplementary material:**

The online version of this article (doi:10.1186/s13063-017-1824-8) contains supplementary material, which is available to authorized users.

## Background

### Background and rationale

Fractures of the upper and the lower extremity with involvement of the joint are, in general, associated with damage to the articular surface. Operative treatment with open reduction and internal fixation is, therefore, recommended for most of these fractures [1–6]. However, the treatment is often complicated by a variable degree of swelling secondary to hemorrhage and soft tissue injury [7–9]. Many of these fractures develop severe swelling that precludes operative intervention until adequate edema resolution has commenced [8, 9]. Patients typically require up to 7 days of inpatient bed rest and elevation of the injured extremity to reduce swelling to an acceptable level for operative treatment with internal fixation [9]. Postoperative swelling of the soft tissue is often responsible for complications, such as compartment syndrome, or local wound problems such as skin necrosis or wound infection [10]. Beside this, the perioperative swelling of the soft tissue is often accompanied by pain leading to prolonged immobilization and the need for strong painkillers with their associated complications. Delay in surgery impacts the patient in the short term but also increases the burden on an orthopedic inpatient trauma unit by prolonging the preoperative interval and total hospital stay [11].

**Fig. 1:**
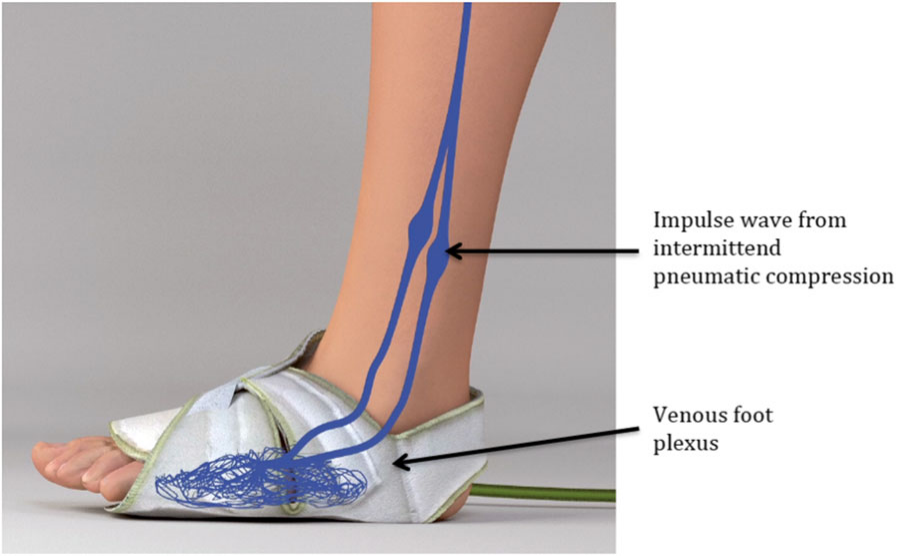
Schematic illustration of the application and the mechanism of the VADOPlex foot-pump system (with kind permission of the Fa. OPED, http://oped-international.com/)

Numerous interventions have been developed to control soft tissue swelling including elevation, compressive dressings and splint immobilization [12–15]. More recently, cryotherapy and intermittent pneumatic compression (IPC) devices have been employed. The IPC devices were first described in the medical literature in 1992 [16]. The main application area of these devices is as antithrombotic therapy, and many reports have been published to prove their antithrombotic effect [16–19]. Beside this, some IPC devices have different pump characteristics but generally tend to rapidly inflate to a pressure of approximately 100 mmHg in less than half a second (Fig. 1). These systems have been shown to stimulate venous return in the immobile lower limb as effectively as walking [20].

Several studies have shown each of these to be effective in reducing edema faster than simple elevation and immobilization [20, 21]. The use of an impulse foot-pump system has previously been shown to reduce edema by means of measurement of ankle and toe girths [10, 11, 20]. The clinical advantages of these devices have been determined in the treatment of ankle fractures and calcaneal fractures [10, 11, 13, 14, 20]. The hospitalization period and surgical site infections were reduced in the patients who received the impulse foot-pump system compared to the control group with elevation. However, the effectiveness of the impulse foot-pump system has not yet been investigated in detail, as complications such as postoperative skin necrosis and compartment syndrome, and the amount of pain medication required have not been determined. Furthermore, data for the impulse foot-pump system are only available for its use in the treatment of ankle and calcaneal fractures.

### Preliminary data

One randomized controlled trial that included 54 patients with ankle fractures compared an impulse foot-pump system with elevation and plaster in the treatment of preoperative swelling [20]. The results of this trial showed a significant reduction in time taken for ankle swelling to settle prior to surgery in the patients using the impulse system, together with a reduction in wound and skin complications (11% versus 44%; *p* < 0.01) and final preoperative ankle swelling (13 mm ± 13 versus 24 mm ± 17; *p* = 0.03). This study was the first to address the issue of whether the impulse foot-pump system has preoperative clinical value in an unselected group of patients with ankle fractures. This study is limited by the fact, that only patients with ankle fractures have been included and that the impulse system was only used in preoperative management. The authors, therefore, concluded that a further randomized controlled trial would be useful to prove the additional benefit in continuing foot-pump therapy in the postoperative phase.

Another study analyzing the effectiveness of the impulse system was published in 2013 [11]; Dodds et al. reported about 64 patients with closed ankle fractures using the impulse system prior to surgery. These patients were compared with a retrospective control group of 73 consecutive patients with closed ankle fractures managed surgically in the same unit immediately prior to the implementation of the impulse device study. The results of this study have shown that the median length of time to surgery, hospital stay duration and surgical site infections were significantly reduced in the study group as compared to the control group. This study is limited by the retrospective nature of the control group. Furthermore, more than half of the patients in the control group and in the study group had an isolated fracture of the medial or lateral malleolus. These fractures are benign regarding soft tissue swelling and do not require special treatment for swelling in general.

There are five more studies available in the medical literature that examined the clinical advantage of impulse foot-pump devices and these were published between 1993 and 2000 [10, 13, 14, 21, 22]. These studies have significant limitations, as only patients with fractures of the ankle and the calcaneus have been included. Even if the impulse foot- or hand-pump systems can be used in fractures of the upper extremity and the tibial head, tibial shaft and tibial pilon, there are no studies available for these anatomical regions. In 2008, Khanna et al. conducted a comprehensive review of the evidence for the use of IPC devices in the healing of fractures and soft tissue injuries [23]. They concluded that the number of subjects in human studies is small and that adequately powered randomized controlled trials in humans are needed to produce stronger evidence that is clinically relevant.

Due to the methodological and clinical limitations of the available studies, a prospective, randomized controlled trial including a variety of injuries that have undergone both pre and postoperative assessment is needed to confirm the observed findings for both the lower and upper extremities. In addition, the design of the impulse foot- and hand-pump system has been renewed (VADOPlex, Vascular Impulse Technology (VIT) system, Fa. OPED, Fig. 1) and data are not yet available for the latest generation of this system.

### Objectives and hypotheses

The primary study parameter is the time to operability of the injured extremity with respect to the surrounding soft tissue. The primary hypothesis of this study is that patients using the VIT system can receive surgical treatment 2 days earlier than patients with elevation due to faster reduction of soft tissue swelling. As mentioned before, the only available data in the medical literature regarding the use of the VIT system pertain to the treatment of ankle and calcaneal fractures. Therefore, the primary study hypothesis can only be investigated in patients with fractures of the ankle, calcaneus and tibial pilon.

## Methods/design

### Design of the study and setting

A prospective, monocenter, randomized controlled trial with a parallel-group design is planned to show superiority of the VIT system compared to elevation of the injured extremity. This study will enroll participants with distinct injury patterns that are associated with severe soft tissue swelling. The study center is a level-I Trauma Center in Germany (BG Trauma Center Ludwigshafen). Trial design and management are the responsibility of the participating trauma center; biostatistical planning and analysis will be performed by the Institute of Medical Biometry and Informatics, University of Heidelberg.

### Screening

Patients with injury of the lower extremity (fractures of the tibial head, tibial shaft or tibial pilon, ankle, calcaneus) or of the upper extremity (fracture of the proximal humerus, distal humerus, distal radius or dislocation of the elbow) that cannot be treated surgically within the first day due to soft tissue swelling will be recruited for this trial. After being informed about the study and its potential risks, all individuals with an appropriate injury pattern will be consecutively screened for eligibility according to the inclusion and exclusion criteria until the recruitment period is over.

### Subject inclusion criteria

- Patients older than 17 years and younger than 81 years of age
- Written informed consent provision
  - One of the following nine injury types with the need for a preoperative inpatient treatment and the need for subsequent open reduction and internal fixation (fractures are classified according to the Comprehensive Classification of the OTA/AO [24]):
    - Intra-articular calcaneal fracture
    - Ankle fracture type 44B/C
    - Tibial pilon fracture type 43B/C
    - Distal radius fracture type 23/C
    - Simple/complex elbow dislocation
    - Distal humerus fracture type 13B/C
    - Proximal humerus fracture
    - Tibial head fracture type 41B/C
    - Tibial shaft fracture

### Subject exclusion criteria

- Patients younger than 18 years or older than 80 years of age
- Injury of the contralateral extremity
- Open fracture/dislocation
- Infection
- Lack of written informed consent
- Severe heart failure
- Acute phlebitis
- Acute thrombosis or pulmonary embolism
- Local problems of the skin (necrosis, bladder)
- Drug abuse
- Serving a gaol sentence
- Pregnancy
- Participation in another interventional trial with interference of intervention and outcome of this study

Patients will be informed about the details of the study. Informed consent comprises a description of the procedures and objectives of the study and the follow-up period. Patients will be informed that participation is completely voluntary. Participation does not confer the patient with any advantages and nonparticipation does not disadvantage the patient in any way. The use by the trial of an already-accredited and CE-certified product will be explained in detail. Potential adverse events regarding the application of the VIT system will also be explained. The participants must provide their consent for the study and for the follow-up. Participants will be instructed to report any exceptional events immediately to the study center. Screening will be performed by the principal investigator or the subinvestigator. A Case Report Form (CRF) will be prepared and will be used for the whole investigation.

### Randomization

For each of the nine injury types a separate randomization to the two intervention groups (VIT group or control group) will be performed. The randomization numbers will be allocated in balanced blocks with varying size (permuted blocks) in a 1:1 ratio using the web-based software “Randoulette” provided by the Institute of Medical Informatics, Statistics and Documentation of the Medical University of Munich (https://wwwapp.ibe.med.uni-muenchen.de/randoulette/index.jsp). This software allows different randomization methods to be chosen as well as different sets of parameters for the chosen method. To avoid any potential of predicting the group allocation of future patients, the block length is recorded in a separate document that is withheld from the study site.

In addition, persons with the right to randomize with the software described above do not have the right to read or edit the randomization design chosen within the software. The software stores the result of randomization and the patient’s characteristics as well as the name of the person who randomized and the randomization date in a separate file, and only authorized persons can download this file. Patients are randomized on the day of inclusion in the study. Blinding of the patients and the assessors is unfeasible. The investigators will perform the assessment according to trial criteria.

### Interventions

The patients included will be randomized in a 1:1 ratio either into the VIT group or into the control group in each of the nine injuries separately. Patients of the VIT group will receive a short instruction regarding the use of the already-accredited and CE-certified VIT system. Afterwards, the VIT system will be applied to the foot in the case of a lower extremity fracture or to the hand in the case of an upper extremity injury. The therapy interval of pneumatic compression will be chosen as recommended by the company: 24 h a day in the preoperative period and 6 to 8 h in the postoperative period. In the control group the injured extremity will be splinted and elevated for 24 h a day in the preoperative period. In the postoperative period the time of elevation is recommended according to the soft tissue swelling. In both groups the duration of the therapy (pneumatic compression or elevation) will be documented during the daily study visits. In all patients, no further antiswelling interventions, such as lymphatic drainage, will be applied. The primary investigator will perform an interim analysis regarding the presence of adverse events and dropouts after the inclusion of the first 30 patients. In case of the occurrence of any adverse event related to the study, the responsible local Ethics Committee will be informed to decide whether the study can by continued or not. No forms of nonignorable clustering regarding the primary outcome parameter have been detected during planning of the study.

### Evaluation

The primary outcome parameter (time to operability) will be assessed day-to-day by one of two independent experienced orthopedic senior consultants, who will define whether the soft tissue permits definite surgical treatment of the fracture, irrespective of other reasons that do not allow surgical treatment. These independent examiners are not involved with the design of the study. The definition of operability is decided by these blinded examiners based on two basic rules: (1) that there is crumpling of the skin at the surgical side and (2) that suturing of the skin without tension will be possible after definitive operative treatment. For the assessment of the primary outcome parameter, the time from injury to the first day of operability will be documented regardless of the time of definitive surgical treatment.

**Fig. 2:**
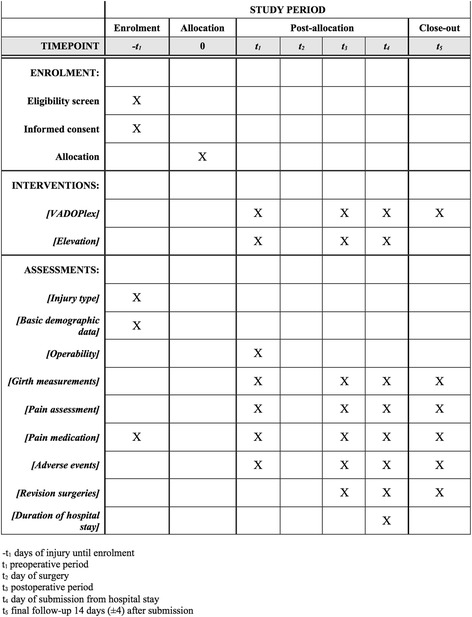
Content for the schedule of enrollment, intervention and assessments for each of the nine injury types (SPIRIT figure)

The time schedule of the study and the primary and secondary outcome parameters and respective measurements are shown in Table 1 and Fig. 2. The final follow-up will be obtained 14 (±4) days after the patients have been formally discharged.

**Table 1:** Please check if we have captured the tables correctly.Study outcome measures of the Vascular Impulse Technology (VIT) study

Item	Outcome measurement	Measurement point	Primary/secondary outcome parameter
Operability	Time from injury to operability (days)	Daily visit preoperatively	Primary
Soft tissue swelling	Girth measurements of the injured and the uninjured contralateral extremity (cm)	Daily visit pre and postoperatively and 14 days after submission	Secondary
Pain level	Visual Analog Scale (VAS)	Daily visit pre and postoperatively and 14 days after submission	Secondary
Pain medication	Substance and dosage of painkillers	Daily visit pre and postoperatively and 14 days after submission	Secondary
Complications	Detailed description of complications	Daily visit pre and postoperatively and 14 days after submission	Secondary
Revision surgeries	Detailed description of revision surgeries	Daily visit postoperatively and 14 days after submission	Secondary
Duration of total hospital stay	Days	At the day of submission	Secondary

### Sample size

The prior assumptions for sample size calculation are based on the results of Caschman et al. and Keehan et al. and assume a decrease of the mean time from injury to operability of 2 days in the VIT group compared to the control group [10, 20]. Allowing for an assumed standard deviation of 2 days and that 15% of the patients in each group will be lost to follow-up then a total of 20 patients (17 patients + 3 patients assumed to dropout) is required in each group to ensure an 80% chance of significant detection (at the 5% level based on a two-sided *t* test). The sample size calculation based on the results of Caschman et al. and Keehan et al. can be used for three of the nine injury types: the calcaneal fracture, the ankle fracture and the tibial pilon fracture. In these three injury types 40 patients will be included, respectively. For the other six injury types a sample size calculation is not possible due to a lack of data in the medical literature. Therefore, in the other anatomical regions a sample size calculation was obtained from the institutional data from the year 2014 (Table 2). It is estimated that 30% of the patients can be recruited after checking the inclusion and exclusion criteria. Taking the institutional data from the year 2014 into account, 30 patients with distal radius fracture, 16 patients with elbow dislocation, 14 patients distal humerus fracture, 30 patients with proximal humerus fracture, 40 patients with tibial head fracture and 30 patients with tibia shaft fracture will be included in the study. The number of patients who will be randomized in each of the nine injury types is shown in Table 2. In total, 280 patients will be included in nine separate studies according to the nine different injury types. The study length for recruitment is expected to be 2 years. The follow-up period is 14 days after discharge from hospital treatment.

**Table 2:** Sample size calculation

Injury	Number of patients in 2014 at the study center	Number of patients included in the planned study
Calcaneal fracture (intra-articular)	119	40^a^
Ankle fractures: type 44B/C	106	40^a^
Tibial pilon fracture: type 43B/C	64	40^a^
Distal radius fracture: type 23C	102	30^b^
Tibial shaft fractures	100	30^b^
Elbow dislocations (simple and complex)	55	16^b^
Distal humeral fractures: type 13B/C	46	14^b^
Proximal humeral fractures	99	30^b^
Tibial head fractures: type 41B/C	138	40^b^
Total		280

^a^sample size calculation are based on the results of Caschman et al. [20]; ^b^sample size calculation based on the treated patients in 2014 in the study center

### Statistical methods

Descriptive statistics (mean, standard deviation, median, interquartile range, absolute and relative frequencies) will be calculated to characterize the study population. A separate analysis for each of the nine injury types will be performed. The primary outcome parameter, the time from injury to operability (in days) will be assessed using a *t* test. In addition, a sensitivity analysis with the nonparametric Mann-Whitney *U* test will be performed. In addition, Kaplan-Meier survival curves for descriptive analysis of the primary outcome parameter will be visualized. For continuous secondary variables a *t* test will be applied, possible differences of categorical endpoints will be analyzed using chi-square tests. A *p* value < 0.05 is considered as statistically significant. Univariate and multivariate regression analyses will be performed to investigate the influence of parameters, such as age, gender and affected side, on the outcome. The primary outcome parameter will be assessed day-to-day by one of two independent experienced orthopedic senior consultants. To validate the agreement of the assessment of the two examiners, the exact agreement regarding the binary variable (operability: yes or not) on 10 consecutive patients will be assessed each day from randomization to definitive surgical treatment. The exact agreement of both examiners regarding the primary outcome parameter should be at least 90%.

The intention-to-treat principle will be adopted: patients will be analyzed according to their initial treatment group in case of crossover to the other group. A sensitivity analysis with a per-protocol analysis will be performed to assess the robustness of the results.

### Handling missing data

The extent of missing data for the primary outcome should be limited, as it is recorded in the daily visits of the study patients. The data entry must be completed electronically in the data management system before discharge and research nurses should make every effort to collect this. Where these time-critical data are missing, multiple imputation methods may be considered to inform a sensitivity analysis.

### Documentation

All protocol-required information collected during the trial must be entered by the investigator, or designated representative, in the Case Report Form (CRF). A paper-based CRF will be used to collect the data. The investigator, or designated representative, should complete the CRF pages as soon as possible after information is collected, preferably on the day of the study visit. Any outstanding entries must be completed immediately after the final examination. An explanation should be given for all missing data.

The completed CRF must be reviewed and signed by the investigator or by an authorized subinvestigator named in the trial protocol. The CRF data will be transferred to an electronic data management system (REDCap). Completeness, validity and plausibility of data are examined by the management system which thereby generates queries. The investigator or the designated representatives are obliged to clarify or explain the queries. At the end of the trial, the principal investigator will retain the originals of all CRFs. The data will be managed and analyzed in accordance with the appropriate standard operating procedures (SOP).

### Assessment of safety

According to the international principles of the Good Clinical Practice (ICH-GCP) the term “adverse event” covers any clinically relevant sign, symptom, syndrome or illness that appears or worsens in a subject during the period of observation in the clinical trial and that may impair the subject’s wellbeing.

Adverse events fall into the categories nonserious and serious. Nonserious adverse events will not be documented in the VIT study. From the beginning of the study until the regular end of trial at 14 days (±4) follow-up or until premature withdrawal of the patient, all serious adverse events (SAE) must be documented on a “Serious Adverse Event Form” that is available in the Investigator Site File. Any other complications that are considered as clinically relevant by the investigator should be documented in free text.

Serious adverse events must be reported by the attending physician to the principal investigator within 1 day after the SAE becomes known. The principal investigator is responsible for registering all SAEs and for checking incoming SAEs for completeness, correctness and plausibility. In the event of an SAE, the principal investigator will inform the local Ethics Committee without delay. Analysis of safety-related data is performed with respect to frequency of SAE in both treatment groups.

### Criteria for termination of the trial

The principal investigator has the right to terminate the trial and to remove all trial material from the trial center at any time in consultation with the trial statistician and the local Ethics Committee. Reasons that may require trial termination include potential health hazards caused by the study intervention as indicated by the prevalence or severity of adverse events, unsatisfactory patient enrollment with respect to quality or quantity, or where data recording is severely inaccurate or incomplete. New external evidence may also necessitate termination of the trial.

### Ethics and trial registration

This study will be carried out according to the Helsinki Declaration in its latest version dated 2004, the Medical Association’s professional code of conduct and the international principles of the Good Clinical Practice (ICH-GCP). The trial will also be carried out in compliance with national legal and regulatory requirements. Additionally, the medical secrecy and the German Federal Data Protection Act will be observed. Patients will receive complete oral and written information about the trial from a physician and a written Informed Consent Form must be signed.

Before the start of the trial, the clinical trial protocol, the Informed Consent Form and any other appropriate documents will be submitted to the Independent Ethics Committee (IEC). The trial was registered in the German Clinical Trial Register (http://www.germanctr.de) with a unique identification number (No. DRKS00010510) on 17 July 2016. Protocol modifications will be made in agreement with the local IEC. The trial management is committed to writing a scientific publication even if the trial is stopped early. The design of the trial and the trial results will be published and the authorship will be assigned by the trial management. Please see Additional file 1 for the SPIRIT Checklist.﻿

## Discussion

If there are two or more treatment options for one clinical condition a randomized controlled trial with a clinically relevant endpoint should determine, which is more beneficial to the patient [25].

Standard therapy for the treatment of preoperative swelling includes inpatient bed rest and elevation of the injured extremity to reduce swelling to an acceptable level for operative treatment [11]. Alternatively, an impulse foot- and hand-pump system (such as the VIT system) can be used to reduce edema of the injured extremity [11, 20]. However, prospective controlled trials are still lacking and the information regarding the effectiveness of the impulse foot- and hand-pump devices is sparse [23].

The proposed study will compare standard treatment and the use of an impulse foot- and hand-pump system (VIT system) for the lower and upper extremities in a prospective, randomized controlled trial setting. In agreement with previous medical literature the time from injury to operability was chosen as primary outcome parameter as this is mainly influenced by preoperative swelling of the soft tissue [10, 20]. The primary study objective of this trial will be to answer the question as to whether using the VIT system will reduce the time from injury to surgery compared to the standard therapy. The results will have a direct implication as preoperative swelling often precludes surgery within the first week after injury. According to the preliminary data from Caschman et al. and Keehan at al. we hypothesize that patients using the Vascular Impulse Technology (VIT) system can receive surgical treatment 2 days earlier than patients with elevation due to faster reduction of soft tissue swelling [10, 20]. Interventions, such as lymphatic drainage, compressive dressings or cryotherapy, have not been added to eliminate the occurrence of selection bias.

A potential study limitation is the risk of performance bias. The primary outcome measurement is based on the assessment of a team of two independent but nonblinded examiners. Even if the assessment is based on prespecified rules, the decision (operability?: yes or no) is primarily based on the experience of the examiner. Therefore, a validation of the exact agreement on the rating of both examiners on 10 consecutive patients will be performed. The primary outcome parameter (time from injury to operability) was chosen for clinical relevance in accordance to previous studies.

### Trial status

The study is about to start enrolling participants.

## Additional file

Additional file 1: SPIRIT 2013 Checklist: recommended items to address in a clinical trial protocol and related documents*. (PDF 113 kb)

## Abbreviations

CRF: Case Report Form
DRKS: German Clinical Trials Register
VIT: Vascular Impulse Technology
